# Research Productivity Among Orthodontic Residents and Fellows Across Training Programs in the United States

**DOI:** 10.7759/cureus.108511

**Published:** 2026-05-08

**Authors:** Alexander Shakhmurov, Valerie Yakubaev, Daniel Nasimov, BS, Tiffany Babakhanov, Alex Rubinov, Avital Priyev-Kalantarov, Matthew Neryaev, Mark M Shimanov, Yanatan Zakinov

**Affiliations:** 1 Biology, Adelphi University, Garden City, USA; 2 Biology, St. John's University, New York City, USA; 3 Biology, Touro University, New York City, USA; 4 Liberal Arts, St. John's University, New York City, USA; 5 Biology, Hofstra University, Hempstead, USA; 6 Dentistry, Jamaica Hospital Medical Center, New York City, USA

**Keywords:** cross-sectional study, dental education, orthodontics, research productivity, residency training

## Abstract

Background: Orthodontic residency programs increasingly expect residents to present research and publish in peer-reviewed journals, yet resident satisfaction with research opportunities remains low. Historical data indicate declining research productivity, but comprehensive current assessments are lacking. Objective: This study aims to quantify and characterize research productivity among orthodontic residents and fellows across training programs in the United States. Materials and methods: Using ADEA Pass, we identified orthodontic residency and fellowship programs and compiled rosters for graduating classes 2025-2028. For each trainee, we recorded demographics and training details and conducted systematic PubMed searches to identify publications. Publications were analyzed for authorship position, article type, timing relative to residency, dental relevance, journal impact factor, and citation counts. Statistical analyses compared productivity by sex and post-graduate year (PGY) status. Results: Among 686 residents and fellows from 42 programs, only 157/686 (23%) had at least one publication, with an average of 0.5 ± 1.8 total publications per trainee. The cohort produced 369 publications, predominantly basic science (152/369, 41%), and published pre-residency (298/369, 81%). Only 73/369 (20%) were first-author publications. PGY 2 residents consistently outperformed residents in other years across all metrics. Males had a higher proportion of dental-related publications than females (114/158 males, 72% vs 135/211 females, 64%; χ²(1, 369) = 3.96, P = 0.029). Comparing final-year residents, longer program duration (3 vs 2 years) did not significantly increase publication output. Conclusions: Most orthodontic trainees have minimal research involvement, with publications concentrated among a few high-output individuals. Despite expectations for scholarly activity, current research productivity suggests inadequate mentorship, resources, or time dedicated to research. Programs must enhance research infrastructure to ensure trainees can critically evaluate and contribute to evolving orthodontic literature.

## Introduction

Research indicates that approximately half of respondents from 62 orthodontic residency programs believe an excellent program should have students who present at meetings and publish in peer-reviewed journals [1]. This expectation aligns with findings from Canadian orthodontic residents, half of whom believe their research requirements should be rigorous enough to warrant a Master of Science degree [2]. Despite these high expectations, resident satisfaction with research opportunities remains problematic. A survey of American orthodontic students found that while research ranked as the least important satisfaction marker, students were nonetheless dissatisfied with their research opportunities. However, 71% of residents still wished to publish their work in peer-reviewed journals [3]. This dissatisfaction has persisted, with recent data showing that half of orthodontic residents remain unhappy with their research mentorship and protected research time [4].

These concerns are reflected in declining research productivity. Historical data revealed a decrease in resident-published papers from an average of 2.4 in 1989 to 1.8 in 1999 [5]. However, current data on orthodontic residents' research output are lacking, making it difficult to assess whether their concerns about mentorship and time are justified. For comparison, a cross-sectional study of maxillofacial surgery residents found each trainee produced approximately 0.43 PubMed-indexed papers [6]. We hypothesize that orthodontic residents may publish slightly fewer papers due to the longer training duration in maxillofacial surgery. Notably, the maxillofacial study did not assess study quality. The purpose of our study is to quantify and characterize the research productivity of orthodontic residents and fellows, providing a comprehensive overview of their research capabilities and interests. Our findings will help educators identify potential gaps in training and validate or refute the concerns expressed in prior resident surveys. Regional bibliometric analyses have documented shifts and declines in orthodontic output over recent decades, underscoring the need to quantify trainee contributions nationally [7].

## Materials and methods

**Figure 1:**
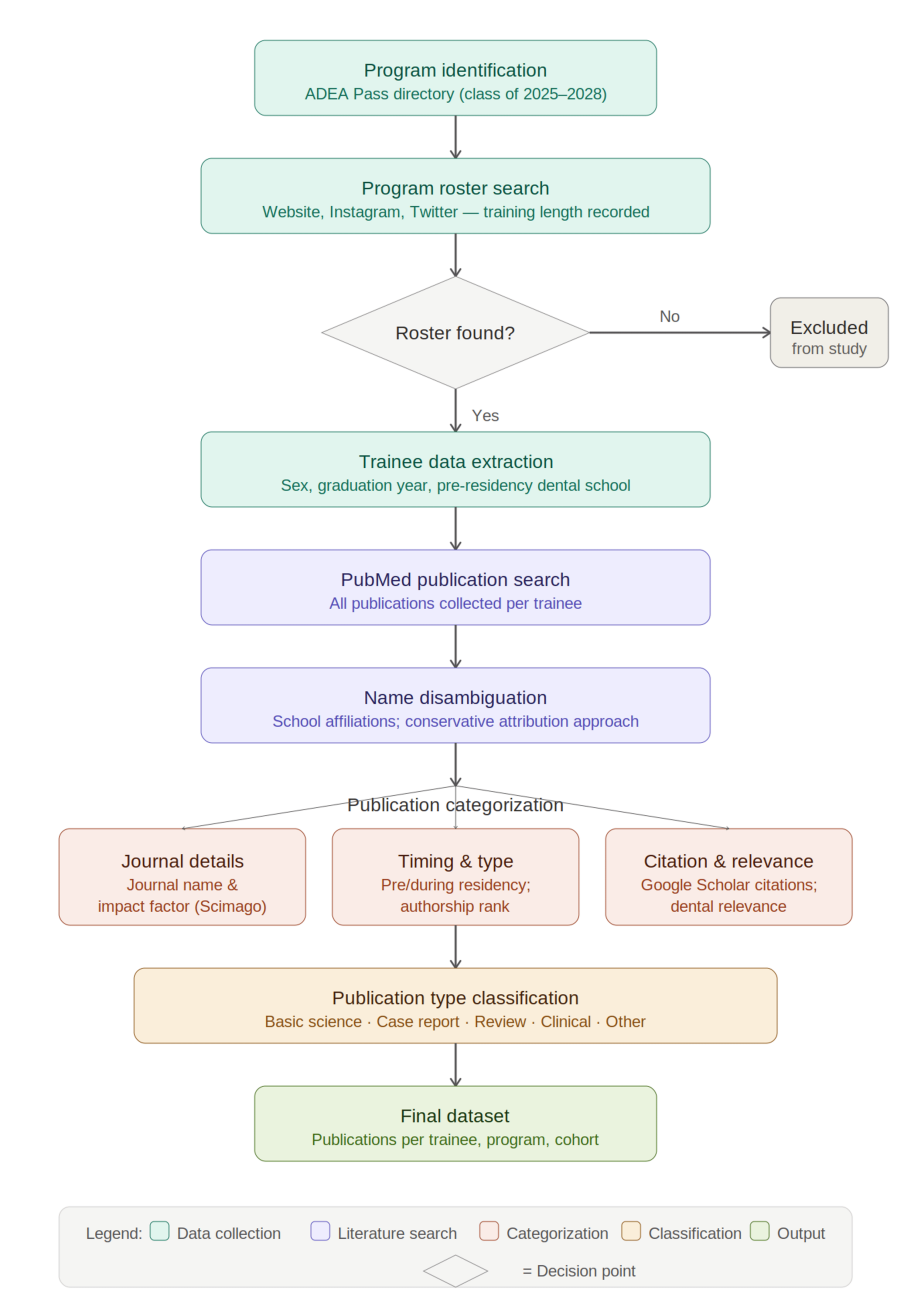
Study design flowchart for analysis of publication output among orthodontic residents and fellows Programs were identified via ADEA Pass and screened for publicly available rosters; those without rosters were excluded. Trainee demographics and PubMed publications were recorded and subcategorized by journal impact factor, publication type, authorship rank, dental relevance, and citation count.

This study used publicly available, de-identified data. Therefore, institutional review board approval and informed consent were not required. A list of all the orthodontic residency and fellowship programs was collected using ADEA Pass [8]. Each program’s website, Instagram page, and Twitter account were searched to identify programs, resident/fellowship rosters (class of 2025-2028), and training lengths (Figure 1). If a roster was not identified, that program was omitted from the study. Each trainee on the roster had their sex, graduation year, and pre-residency dental school recorded. Following this, the trainees' names were entered into PubMed (first name, last name), and all publications by each trainee were collected until December 22, 2025. Generic names (e.g., John Smith) were scrutinized for affiliations with undergraduate, dental, or orthodontic schools. If a person with a generic name had one or more manuscript-listed matching affiliations based on their profile listed on their program's student roster, then that paper was included in the dataset. A conservative approach was taken to not over-attribute publications to these individuals. Each student’s publications were further subcategorized by journal, and the journals' impact factors were quantified using Scimago [9].

Furthermore, the following was collected: work accepted for publication before or during residency/fellowship; publication type; the number of times the work was cited on Google Scholar [10]; the publication's relevance to dentistry; and authorship rank. Publication types were assessed through a review of the paper’s abstract and categorized as basic science, case report, review, clinical (translational), or “other” articles that do not fit the aforementioned descriptions. The publication types were ascribed using two reviewers for each article. It was determined that if two reviewers disagreed on the categorization of an article type, a third reviewer would decide its category.

Descriptive data were calculated for the whole cohort. Stratifications were made based on sex and PGY status. Publication metrics were presented as total counts, with summary statistics as mean ± standard deviation and categorical statistics as frequencies and percentages. Percentiles for major publication statistics were calculated at the 10th through 90th percentile in 10% intervals (non-inclusive). Chi-square tests were used to compare males and females, including proportions with at least one publication, proportions with 1st-author publications, and distributions of article types (e.g., basic science and clinical studies). The same comparisons were made comparing PGY 1, PGY 2, and PGY 3 students. To compare pre-residency production by PGY status for total publications and first-author publications, an analysis of variance (ANOVA) was used. If the overall ANOVA was significant, post-hoc Tukey’s HSD test was used to perform pairwise comparisons. An independent-samples t-test was used to compare total publications between second-year students in two-year programs (24 months or less) and third-year students in three-year programs. P-values < 0.05 were considered statistically significant. All analyses were performed in SPSS Statistics version 27.0 (IBM Corp. Released 2020. IBM SPSS Statistics for Windows, Version 27.0. Armonk, NY: IBM Corp.).

## Results

This study included 686 residents and fellows from 42 of 69 residency and fellowship programs. Most trainees were female (404/686, 59%), with the remainder of the cohort consisting of 280 males (280/686, 41%) and two unknowns (2/686, <0.1%). Among those included, 276/686 (40%) were PGY 1, 220/686 (32%) were PGY 2, 171/686 (25%) were PGY 3, 12/686 (2%) were fellows, and 7/686 (1%) were unknown PGY. Columbia University College of Dental Medicine contributed the greatest number to the cohort (n = 29), followed by University of Pennsylvania School of Dental Medicine (n = 22) and University of California, San Francisco School of Dentistry (n = 22), with 64 other dental schools contributing at least one resident/fellow. International dental schools produced 119 residents and fellows in the cohort. All these students were residents or fellows across 42 programs, with the Georgia School of Orthodontics - Hudson Regional Hospital contributing the greatest number (123/686, 18%). Residency program lengths varied, but most included programs were 36 months (12/42, 29%).

There were 369 total publications included in this analysis. PGY 2 residents (163/369, 44%) and females (211/369, 57%) contributed the greatest amount. A minority of publications had the resident/fellow as the first author (73/369, 20%). Most of the papers were published prior to residency (298/369, 81%). Basic science papers were most common (152/369, 41%). All publications combined had 9,489 citations, with an average journal impact factor of 1.1 (Table 1).

**Table 1:** Orthodontic residents and fellows publication metrics People with unknown PGY and sex were only included in the overall category. Percentages indicate the proportion compared to total publications in that subgroup. PGY: post-graduate year

Count	Overall (n = 686)	PGY 1 (n = 276)	PGY 2 (n = 220)	PGY 3 (n = 171)	Fellows (n = 12)	Male (n = 280)	Female (n = 404)
Total publications	369	111	163	85	6	158	211
First author	73 (20%)	24 (22%)	37 (23%)	11 (13%)	1 (17%)	31 (20%)	42 (20%)
Second author	83 (22%)	21 (19%)	39 (24%)	22 (26%)	1 (17%)	42 (27%)	41 (19%)
Before residency	298 (81%)	98 (88%)	137 (84%)	60 (71%)	0 (0%)	121 (77%)	177 (84%)
During residency	71 (19%)	13 (12%)	26 (16%)	25 (29%)	6 (100%)	37 (23%)	34 (16%)
Dentistry related	249 (67%)	74 (67%)	109 (67%)	56 (66%)	6 (100%)	114 (72%)	135 (64%)
Basic science	152 (41%)	43 (39%)	72 (44%)	37 (44%)	0 (0%)	65 (41%)	87 (41%)
Clinical study	99 (27%)	35 (32%)	36 (22%)	23 (27%)	1 (17%)	38 (24%)	61 (29%)
Review article	70 (19%)	22 (20%)	36 (22%)	10 (12%)	2 (33%)	24 (15%)	46 (22%)
Case report	19 (5.1%)	2 (1.8%)	11 (6.7%)	6 (7.1%)	0 (0%)	13 (8.2%)	6 (2.8%)
Total lifetime citations	9,489	1,873	4,563	2,909	139	3,688	5,801
Average impact factor	1.1	1.2	1.0	1.1	1.0	1.0	1.1

The publications were found across 226 unique journals, with the American Journal of Orthodontics and Dentofacial Orthopedics having the most relevant publications (n = 12). Three journals tied for second place with six such publications (BMC Oral Health, Bone, and Angle Orthodontist).

The cohort averaged 0.5 ± 1.8 total publications, of which 0.1 ± 0.5 were first-author publications. The most publications for any one student were 30, but even having just a single publication would place a student in the 80th percentile (Table 2).

**Table 2:** Percentile breakdown of publication metrics for orthodontic residents and fellows x̄ = mean ± standard deviation IF: impact factor

Percentile	Total x̄ = 0.5 ± 1.8	First author x̄ = 0.1 ± 0.5	Before residency x̄ = 0.4 ± 1.6	Basic science x̄ = 0.2 ± 1.3	Clinical x̄ = 0.1 ± 0.5	Dentistry-related x̄ = 0.4 ± 1.1	Average IF x̄ = 0.2 ± 0.5	Total citations x̄ = 13.8 ± 74.9
Maximum	30	7	26	27	5	11	5.18	920
90th	2	0	1	1	0	1	0.88	15
80th	1	0	0	0	0	0	0.39	1
70th	0	0	0	0	0	0	0	0
60th	0	0	0	0	0	0	0	0
50th	0	0	0	0	0	0	0	0
40th	0	0	0	0	0	0	0	0
30th	0	0	0	0	0	0	0	0
20th	0	0	0	0	0	0	0	0
10th	0	0	0	0	0	0	0	0
Minimum	0	0	0	0	0	0	0	0

Comparing male and female students across PGY levels showed minimal differences. Both male and female students: PGY 2s consistently outperformed PGY 1s and PGY 3s across all major metrics (Table 3).

**Table 3:** Average and median publication metrics categorized by sex and PGY for orthodontic residents and fellows IF: impact factor, PGY: post-graduate year, SD: standard deviation

	Male (n = 280)				Female (n = 404)			
Mean ± SD	PGY 1	PGY 2	PGY 3	Fellow	PGY 1	PGY 2	PGY 3	Fellow
Total publications	0.4 ± 1.0	0.8 ± 1.8	0.6 ± 1.7	1.2 ± 2.7	0.4 ± 1.4	0.7 ± 2.9	0.4 ± 1.0	0.0 ± 0.0
First author	0.1 ± 0.4	0.2 ± 0.8	0.1 ± 0.3	0.2 ± 0.5	0.1 ± 0.4	0.2 ± 0.7	0.1 ± 0.3	0.0 ± 0.0
Before residency	0.3 ± 0.7	0.6 ± 1.5	0.4 ± 1.6	0.0 ± 0.0	0.4 ± 1.4	0.6 ± 2.6	0.3 ± 0.8	0.0 ± 0.0
Basic science	0.1 ± 0.6	0.3 ± 1.1	0.3 ± 1.2	0.0 ± 0.0	0.2 ± 0.8	0.4 ± 2.4	0.1 ± 0.4	0.0 ± 0.0
Clinical	0.1 ± 0.4	0.2 ± 0.5	0.1 ± 0.4	0.2 ± 0.4	0.1 ± 0.6	0.2 ± 0.5	0.1 ± 0.4	0.0 ± 0.0
Dentistry related	0.3 ± 0.8	0.6 ± 1.6	0.4 ± 0.9	1.2 ± 2.7	0.3 ± 1.0	0.5 ± 1.4	0.3 ± 0.7	0.0 ± 0.0
Average IF	0.2 ± 0.6	0.3 ± 0.7	0.2 ± 0.5	0.2 ± 0.5	0.2 ± 0.6	0.2 ± 0.4	0.2 ± 0.5	0.0 ± 0.0
Total citations	5.3 ± 25.8	14.9 ± 53.0	21.0 ± 96.0	27.8 ± 62.2	7.7 ± 60.1	24.8 ± 115.4	13.8 ± 68.2	0.0 ± 0.0

**Figure 2:**
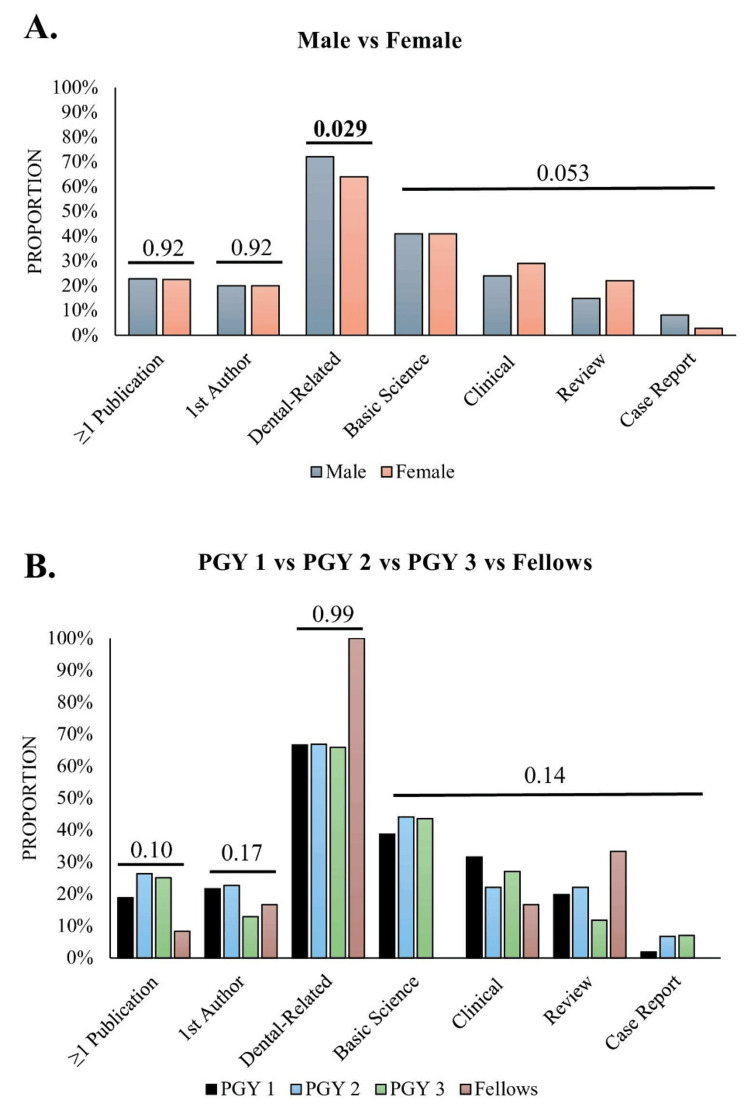
Comparison of publication metrics among orthodontic residents by sex (A) and PGY (B) The value above the bars displays the p-value for the comparison. Bolded values indicate P < 0.05. The fellowship group was not included in comparisons, as only one student contributed to all the publications in that group. Proportions for the "≥1 publication" category indicate the proportion of students with at least one publication within the whole subgroup. The remaining categories refer to the proportion of publications contributed by that group that meet categorization criteria relative to the total publications in that group.

Comparing male and female students based on the proportion with at least one publication did not show a difference between the groups (64/280 males, 23% vs 93/404 females, 23%; χ²(1, 684) = 0.003, P = 0.92). There were also no sex-based differences in the proportion of first author publications (31/158 males, 20% vs 42/211 females, 20%, χ² (1, 369) = 0.003, P = 0.92). Males had a greater proportion of dental-related publications (114/158 males, 72% vs 135/211 females, 64%, χ² (1, 369) = 3.96, P = 0.029), but there were no sex-based differences in article type (χ² (3, 369) = 7.80, P = 0.053) (Figure 2A). There were no differences in these metrics across students by PGY status. However, among the 12 fellows, only 1/12 (8%) contributed any publications (Figure 2B).

**Figure 3:**
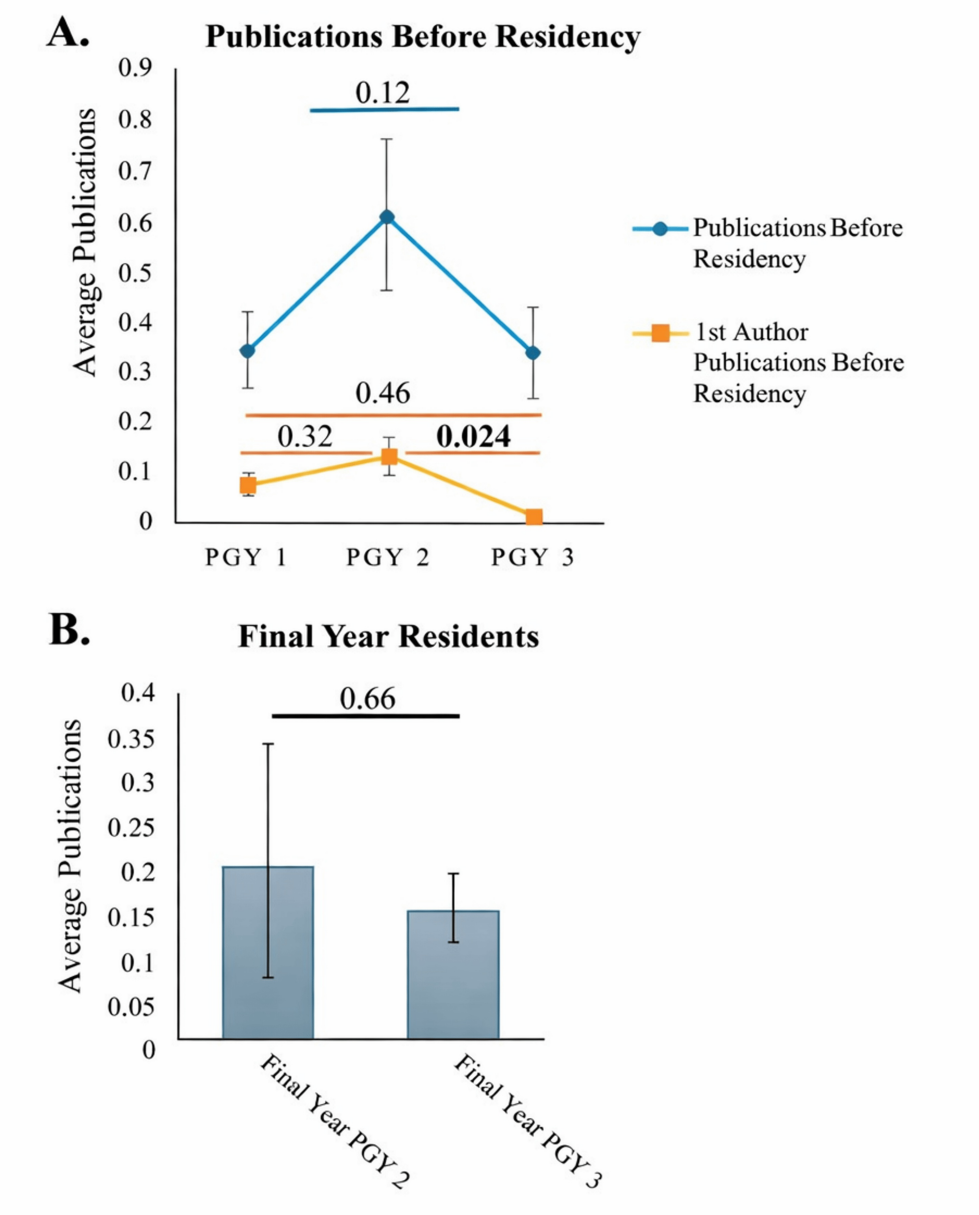
Comparisons of pre-residency publications stratified by PGY (A) and comparison of publication quantity for final year orthodontic residents in programs greater and less than two years (B) Values above the lines indicate p-values. In A, pairwise comparisons were only performed if the overall ANOVA was significant (as was the case for the first author's publications). Error bars represent the standard error of the mean. PGY: post-graduate year, ANOVA: analysis of variance

Comparisons of pre-residency production across the three years of students are shown in Figure 3A. While not statistically significant (F (2, 667) = 1.2, P = 0.12), the PGY 2 year had a seemingly higher number of publications pre-residency (PGY 2: 0.6 ± 2.2 vs 0.4 ± 1.2 for both PGY 1s and PGY 3s). However, when comparing the average number of first-author publications before residency, differences emerged (F(2, 667) = 3.1503, P = 0.044). The PGY 2 cohort had significantly more first-author publications than the PGY 3 cohort (0.1 ± 0.7 vs 0.02 ± 0.3, P = 0.024). There were, however, no statistically significant differences between the PGY 2 cohort and the PGY 1 cohort (0.1 ± 0.7 vs 0.07 ± 0.4, P = 0.32). Comparisons of PGY 2 and PGY 3 students in their final residency year (two- and three-year programs, respectively) showed that total publication output did not differ significantly between the groups. However, the cohort with the shorter residency had more publications on average (PGY 2 in final year: 0.2 ± 0.6 vs PGY 3 in final year: 0.1 ± 0.5; t(190) = 0.45, P = 0.66) (Figure 3B).

## Discussion

The present study sought to quantify the research production of orthodontic residents and fellows. Given that understanding advances in research is essential to eventual clinical practice, students with experience conducting such research may be better equipped to navigate and interpret the existing literature. This is particularly important because research in the dental industry is highly concentrated among a minority of teaching institutions [11]. Therefore, many students may experience little to no scholarly involvement throughout their general dental education and specialty training. The present study found that a large majority of orthodontic residents and fellows had no publications at the time of data collection. This may indicate that many orthodontic trainees have little exposure to the research process. Having familiarity with the research process is a vital aid for assessing the efficacy of the literature they may use to improve their practice. Therefore, there is good reason to be concerned that, given the lack of formal research involvement (through all stages, particularly in publishing), future orthodontists may be ill-equipped to stay up to date with current best practices [12].

Only about one in five residents and one in twelve fellows in this study had published. This contrasts with a recent study of oral maxillofacial surgery (OMS) residents, in which about one in two had at least one publication [6]. However, it is important to note that this OMS study is not directly comparable due to the longer residency. Despite this, the average number of publications among orthodontic students (0.5) in this study was actually greater than that among OMS students (0.43) [6]. This indicates that publications among orthodontic residents/fellows are highly centralized to a small subset of high-output research performers. In general, the amount of orthodontic literature produced in the United States has been decreasing over the past decade (2011-2020) [13]. Therefore, there is likely a need for greater student and resident involvement to ensure the literature remains up to date. Encouragingly, the PGY 2 class included in this study had significantly more pre-residency publications than the PGY 3 class, suggesting a trend toward greater research involvement in upcoming classes. However, this may have been a normal yearly variation, as the trend did not continue into the PGY 1 class, which produced publication numbers similar to those of the PGY 3 class before residency. The lack of increased research involvement before residency stands in stark contrast to developments in medical degree programs. Across virtually all medical specialties, the number of student-led/involved publications has increased dramatically [14,15]. Promisingly, though, is that the orthodontic residents/fellows in this study contributed most to basic science papers, a finding not true of medical students. Basic science research tends to take the longest to publish and is perhaps the best way to learn the scientific principles that govern research and scholarship [14]. Therefore, the ultimate goal should not be to increase the average quantity of research, but rather to ensure that all students (whether in dental school or residency) are involved in meaningful research. Furthermore, resident/fellow research involvement was primarily in dentistry-related areas, which provides exposure to literature relevant to their future practice.

Comparisons between male and female residents/fellows did not show major differences. While males had a larger proportion of dental-related publications, the true difference was not substantial. Additionally, comparing by PGY status did not reveal major differences. This indicates that only a small number of research papers were published during residency. Furthermore, for students in their final year of residency, an additional year of residency (3 vs 2) did not significantly alter the average number of publications.

Residency would be an ideal time to conduct research, as students have narrowed their focus, and a research project would enable them to further engage with the literature in their chosen field and contribute to its growth. However, residency also presents unique challenges that may prevent, or at least discourage, students from engaging in research. These factors may include a lack of resources (faculty involvement, biostatistical training or support, and funding) and the obvious time constraints [15]. Empirical studies of research conduct in higher education identify mentorship, training in research methods, and institutional incentives as recurring barriers that likely contribute to low trainee publication rates [16]. Given the value of meaningful research involvement in training and learning, programs without a research foundation can find creative ways to encourage research involvement among their residents and fellows. This may include forming partnerships with local universities, providing research mentors, and allocating dedicated research time [17]. Overall, these are possible systemic barriers that may affect research quantity and quality amongst students, which are topics for future research investigation.

This study is not without limitations. Firstly, there is inherent selection bias since some programs do not list their student rosters. Additionally, research may take time to be published; therefore, publications included in this study do not represent all the manuscripts that these residents/fellows may have contributed to. Furthermore, not all research endeavors result in a publication, so resident/fellow research involvement may have been greater than these results suggest. Common names may have contributed to the difficulty ascribing certain publications to students. While all efforts were made to ensure correct matching (e.g., confirming affiliations), there may have been errors in this process. The data in this study cannot quantify the richness of the learning experience associated with each publication. Therefore, it is not reasonable to assume that greater involvement in research is equivalent to better education. Lastly, because articles needed to be PubMed-indexed to ensure a certain level of quality, this would exclude non-PubMed-indexed articles from search results.

## Conclusions

Most orthodontic residents/fellows do not contribute to scholarly publications at any point in their training. Increasing residency length does not appear to correlate with greater publication output. While the PGY 2 class produced the most publications, there was little overall difference in pre-residency research output across PGY status. Therefore, there does not seem to be a trend toward increasing publication numbers, as in the medical field. While much of the published research was basic science, which offers significant learning opportunities, the limited involvement of residents/fellows in research is concerning. With the recent decline in orthodontic literature, the lack of involvement in current orthodontic training is expected to worsen this trend. Programs and dental schools must find ways to increase faculty mentorship, provide research opportunities, and allocate dedicated time for research to ensure their trainees can understand the continually evolving literature and contribute to it.
